# Analysis and Reporting of Randomized Trials in Cleft Palate Surgery: Learning from the Timing of Primary Surgery (TOPS) Trial

**DOI:** 10.1177/10556656241253949

**Published:** 2024-05-09

**Authors:** Matthew Fell, Ginette Phippen, Stephanie van Eeden, David Chong, Marc C. Swan, Simon van Eeden, John B. Carlin

**Affiliations:** 1 The Cleft Collective, University of Bristol, Bristol, UK; 2 The Spires Cleft Centre, Oxford University Hospitals NHS Foundation Trust/Salisbury NHS Foundation Trust, Oxford, UK; 3 School of Education, Communication and Language Sciences, 5994 Newcastle University, Newcastle upon Tyne, UK; 4 Plastic and Maxillofacial Surgery, The Royal Children's Hospital, Melbourne, Australia; 5 Alder Hey Children's and Aintree University Hospitals, Liverpool, UK; 6 34361 Murdoch Children's Research Institute, Melbourne, Australia; 7 Melbourne School of Population and Global Health, University of Melbourne, Melbourne, Australia

**Keywords:** assessment, cleft palate, evidence-based practice

## Abstract

The Timing of Primary Surgery (TOPS) trial was published August 2023 in the New England Journal of Medicine and is a milestone achievement for a study focused on cleft palate. Due to the complexity of outcome reporting in cleft and the rarity of such comparative trials, TOPS presents a useful opportunity to critically review the design, analysis and reporting strategies utilised. This perspective article focused on the inclusion of participants, the choice of the primary outcome measure and the analysis of ordinal data within the trial. Considerations for future comparative studies in cleft care are discussed.

## Overview

The Timing of Primary Surgery (TOPS) trial was reported by Gamble et al.^1^ on 31^st^ August 2023 in the New England Journal of Medicine (NEJM) and is a milestone achievement for a study focused on cleft palate. TOPS was an international (5 countries), multi-site (23 clinical sites), parallel-arm randomised trial and was a unique international collaborative effort involving multiple disciplines and many years of planning and execution. The trial was notable in the field of cleft due to the large number of participants (558), impressive retention rate at 5-year follow-up (approximately 85% for viable speech outcomes), cross linguistic analysis and the considerable efforts taken to strive for standardised surgical intervention and speech data collection.^2^ Given the enormity of the TOPS endeavour, the largest cleft trial to date, there was considerable expectation for the delivery of a definitive conclusion.^3^

The aim of the trial was to determine whether it is better to perform cleft palate reconstruction at 6 or 12 months of age, by randomly assigning patients to initial surgery at each age, and assessing outcomes in speech, hearing, dentofacial development and safety (peri-operative adverse events and post-operative complications). The prespecified primary endpoint was velopharyngeal insufficiency (VPI) at 5 years of age, which was defined as a velopharyngeal composite summary (VPC-Sum) score of at least 4.^4^ The headline finding was a reduced prevalence of VPI at 5 years of age of 9% in the 6-month group compared to 15% in the 12-month group (risk ratio 0.59, 95% CI 0.36–0.99; p = 0.04). This was the basis of the authors’ conclusion that infants undergoing surgery at 6 months of age were less likely to have VPI at age 5 than infants undergoing surgery at 12 months of age.^1^

The accompanying editorial by Tse and Jackson gave a balanced discussion of the TOPS trial and advised caution for the interpretation of the trial data when considering the implications for surgical practice.^5^ One of the stated reasons for this caution was that the primary endpoint at 5 years reflected the mixed effects of both primary palatoplasty and secondary speech surgery. With a higher proportion of secondary speech surgery in the 6-month group, it is possible that the improvement in VPI reported in the TOPS trial publication is explained at least in part by the greater use of secondary surgery in that group. The editorial also cautioned to consider the risks of earlier surgery in infants, when many outcomes, such as midfacial growth, will only become apparent at a later age.

Due to the complexity of outcome reporting in cleft^6–9^ and the rarity of such comparative trials,^3,10^ TOPS presents a useful opportunity to critically review the design, analysis and reporting strategies utilised. In this article we focus on areas of the trial that warranted further discussion than the trial itself or the accompanying editorial could afford.^5^ Our overall aim is to consider the ways in which the TOPS data can be interpreted beyond a simple dichotomous declaration about the primary endpoint and to generate a discussion about future comparative studies in cleft care.

## Relevant Population

The applicability of the TOPS trial is related to the participants that were included. The inclusion criteria resulted in a large proportion of children born with a cleft palate (1331/1889: 70%) being excluded. The most common reason for exclusion was an associated syndrome or developmental delay (37% of exclusions). Children born with cleft palate only are known to have a higher prevalence of syndromes compared to other orofacial cleft subtypes, so the exclusion of children with syndromes means that the findings from TOPS cannot be applied to a significant proportion of patients born with a cleft palate.^11,12^ Another common reason for exclusion was infants being deemed not medically fit for an operation at age 6 months by the operating surgeon (36% of exclusions), yet this is a subjective decision without specified parameters, and therefore opens susceptibility to individual surgeon variation. Exclusion due to the cleft palate being too wide for closure with the Sommerlad technique in a single stage (7.4% of exclusions) is an additional subjective surgical decision, which led to a median width of cleft palate at the hard-soft palate junction of 7.0 mm (IQR 4.0 to 9.0 mm) amongst the included participants in both arms of the trial. Overall, this highly selected group of medically fit, non-syndromic children born with a narrow cleft of the palate only receiving surgical intervention in a high-income setting was feasible for the trial design but does not represent the real-life population presenting to global cleft teams, in which the effect of age at primary surgery may be different.

## Choice of the Primary Outcome Measure

The primary outcome (VPI at 5 years of age) in the TOPS Trial was assessed by two speech outcome measures: the primary measure was the Velopharyngeal Composite Summary (VPC-Sum) and the secondary measure was the Velopharyngeal Closure Rating (VPC-Rate, although the abbreviation of ‘rate’ from ‘rating’ is a misnomer). These are potential sources of confusion as both are auditory perceptual judgements of speech and have demonstrated convergent validity with moderate correlation.^13^ It is important for the interpretation of the trial to understand what these outcome measures are and how they are different.

The primary measure of VPI in the trial was based on the VPC-Sum score, initially developed for the Scandcleft trials.^14,15^ The VPC-Sum is derived from listening to single word tests and therefore was amenable to cross-linguistic analysis by three speech therapists with different language backgrounds. VPC-Sum has been recommended as an additional choice to VPC-Rate, for the perceptual assessment of VPI in research settings.^13^ VPC-Sum is a 7-point ordinal scale with scores ranging from 0–6 and it incorporates three elements: hypernasality, non-oral errors and velopharyngeal insufficiency symptoms (including nasal airflow errors). For the primary outcome, the range of VPC-Sum scores was dichotomised into a binary outcome measure to denote sufficient versus insufficient velopharyngeal function and this decision was published a priori.^4^ This gave rise to the headline effect estimate, which provided moderate evidence of a difference in the primary endpoint between the trial arms.

The secondary measure of VPI in the trial was obtained from the VPC-Rate. This provides an assessment of velopharyngeal competence based on listening to continuous speech. The VPC-Rate has been recommended as a first-line clinical choice in the perceptual assessment of VPI in both clinical and research settings.^13^ VPC-Rate is reported as a 3-point ordinal scale (sufficient, marginal and insufficient) but was dichotomised in the trial into a binary outcome of whether the child had ‘insufficient’ velopharyngeal function.^11^ In the TOPS trial, the VPC-Rate scores showed no evidence for a difference between the proportion of children rated to have insufficient velopharyngeal function (8.9% for the 6-month group vs 9% for the 12-month group, RR 0.98, 95% CI 0.55 to 1.76; p = 0.95).

The use of multiple perceptual speech outcome measures for the primary outcome of VPI makes for complex interpretation and raises an important question of whether the VPC-Sum should have been prioritised as the primary endpoint. The VPC-Sum used selected words to pose a challenge to cleft speech characteristics whereas the VPC-Rate used continuous speech, which is a more ecologically valid measure of the child's natural level of speech.^16–18^ There are advantages to both approaches but there is no consensus about which type of measure is superior for the scenario in TOPS, where both velopharyngeal function and articulation were assessed across multiple languages. The way in which the trial was reported gave a higher profile and weighting to the VPC-Sum outcome compared to the VPC-Rate and created an illusion of certainty. A broader consideration of the outcomes reported in TOPS suggests an interpretation that is less clear cut.

## Analysis of VPC-Sum Ordinal Data

Ordinal scales are non-numeric, ranked outcomes, where the distances between the categories are not necessarily meaningfully quantifiable or equally spaced.^19^ Ordinal outcomes are commonly used in healthcare (eg, Glasgow Coma Scale and Rankin Score) but can be difficult to interpret and can be analysed in multiple ways. For ease of interpretation, ordinal scales are often dichotomised by being forced into two categories via a more or less arbitrary cut-point. However, this is potentially misleading because the derived effect estimate will only reflect the specific cut-point and may not provide an overall summary of the data.^20^

The 7-point ordinal scale of the VPC-Sum was dichotomised in TOPS to denote sufficient (score 0–3) versus insufficient (score 4–6) velopharyngeal function and to our knowledge, this is the first time it has been analysed in this way. In comparison, in earlier research the validity of the VPC-Sum was scrutinized by pooling the VPC-Sum scores into three levels to denote sufficient (score 0–1), borderline (score 2–3) and insufficient (score 4–6) velopharyngeal function.^13^ This three-tiered summary of VPC-Sum scores was used in the Scandcleft trials,^15^ yet has also been questioned because of the potential for mild hypernasality, in the absence of other symptoms, to be categorised as ‘sufficient’ velopharyngeal function.^21^ The dichotomisation of the VPC-Sum scores in the TOPS trial raises the potential for even greater degrees of hypernasality severity to be categorised as ‘sufficient’ velopharyngeal function (score 0–3). The ideal cut point in the VPC-Sum ordinal scale to distinguish between the absence and presence of VPI is debateable.

An alternative approach to ordinal data are ordinal regression models, which may provide a more sensitive analysis by producing a summary effect measure that uses information across all of the ordinal categories.^22^ The proportional odds model is increasingly being utilised in clinical trials and is based on the assumption that the treatment effect, measured as the odds ratio relating to the probability of being above (or below) each point on the scale, is constant across the sequence of cut-points.^22^ For the VPC-Sum data in TOPS, the proportional odds assumption provides a summary effect estimate (odds ratio) that represents the relative odds of having a worse outcome (ie, evidence of VPI at 5 years) in the 6-month group compared to the 12-month group, independent of the level of severity used to classify VPI.

**Figure 1.**
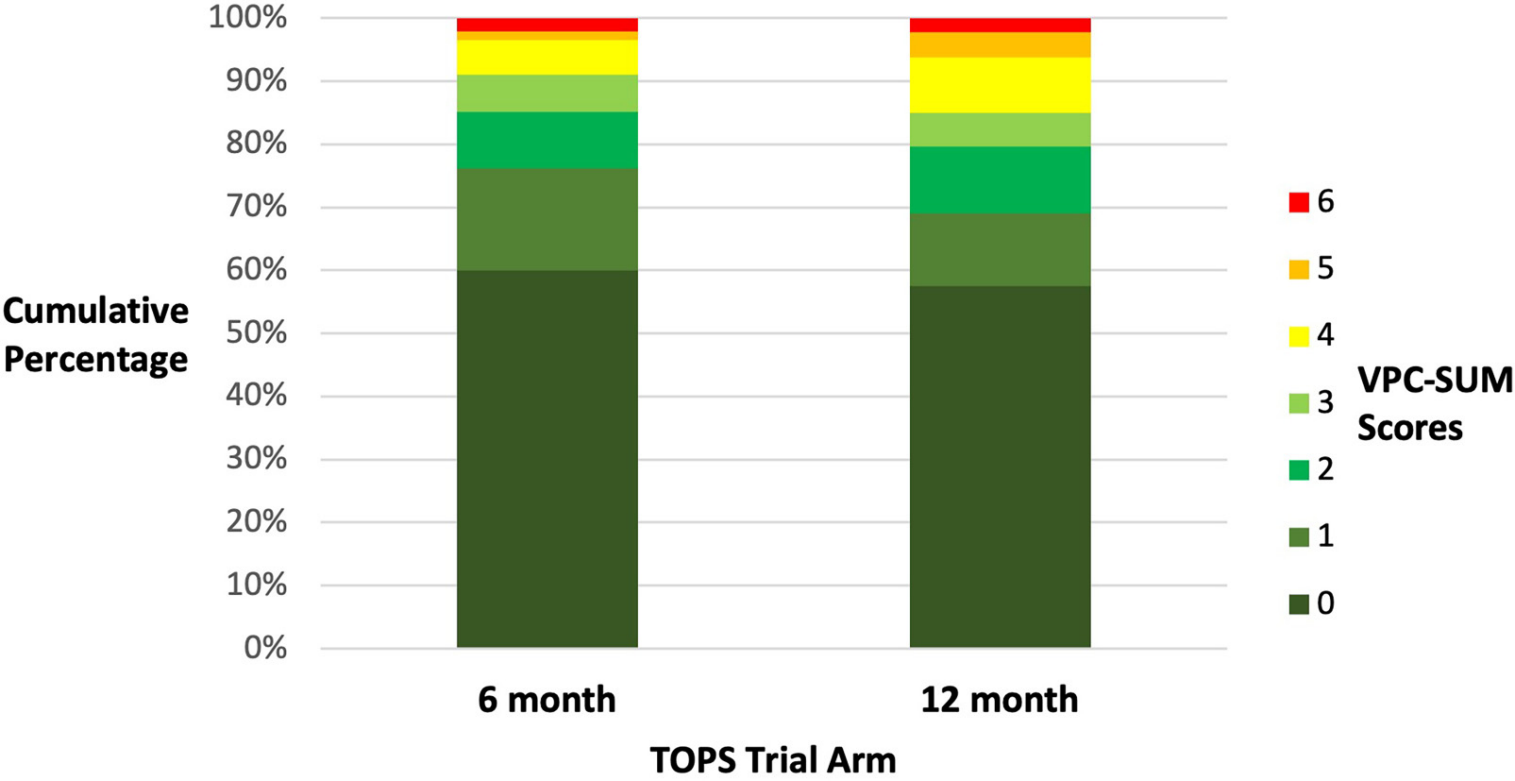
Bar chart showing the cumulative percentages for the VPC-sum ordinal scores at 5 years of age for the 6-month and 12 month surgical groups.

We obtained the raw VPC-Sum 7-point ordinal data through a data request from the TOPS Trial Group as this was not reported in the trial publication (see Figure 1 and supplementary data). First, we estimated the risk ratio (intervention effect) at each of the 6 possible cut-points on the ordinal scale defined by the 7 VPC-Sum categories. Second, we estimated a single overall effect measure as a summary odds ratio under a proportional odds assumption, using an ordinal logistic regression model (See Table 1 and Supplementary data for Stata Code). Whilst there is general consistency in terms of the direction of the effect estimates, it is notable that the only scenario in which the corresponding null hypothesis test meets the conventional level of statistical significance is when the cut-point is set between VPC-Sum score 3 and 4, as was done for the primary analysis of the trial, as pre-specified in the statistical plan. This provides a salient reminder of the tenuousness of dichotomous conclusions that rely on a p-value threshold of significance.^23^

**Table 1.** Alternative Analyses of VPC-Sum Ordinal Data: Dichotomising the Ordinal Data at 6 Potential Cut-Points, with Corresponding Estimated Risk Ratio and P-Value by Standard Chi-Squared, Versus Analysis Assuming Proportional Odds Across all Levels of the Ordinal Scale, Using Ordinal Logistic Regression to Estimate Summary Odds Ratio with Confidence Interval and P-Value.

	VPC-Sum Score	6-month group	12-month group	Effect Estimate	95% CI	P Value
Choice of cut point:						
0|1	0					
	1–6	40%	42%	RR 0.94	0.76 to 1.17	0.59
1|2	0–1					
	2–6	24%	31%	RR 0.77	0.57 to 1.04	0.09
2|3	0–2					
	3–6	15%	20%	RR 0.73	0.49 to 1.09	0.12
3|4*	**0–3**					
	**4–6**	**9%**	**15%**	**RR 0.59**	**0.36 to 0.99**	**0**.**04**
4|5	0–4					
	5–6	3%	6%	RR 0.55	0.24 to 1.28	0.16
5|6	0–5					
	6	2%	2%	RR 0.96	0.28 to 3.28	0.95
Ordinal analysis^β^	0	60%	58%	Summary OR 0.82	0.57 to 1.16	0.26
	1	16%	12%			
	2	9%	11%			
	3	6%	5%			
	4	6%	9%			
	5	1%	4%			
	6	2%	2%			

*This is the cut-point used for the primary analysis of Gamble et al.^1^ and was chosen a priori.^4^

β The Brant Test was used to assess the validity of the proportional odds assumption on this data. The p value of 0.29 suggests the data is compatible with the proportional odds assumption.

There is no necessarily ideal way to analyse ordinal data,^19^ so the purpose of this exercise of exploring various analytic scenarios is to emphasise the range of conclusions that might be drawn depending on the definition of the treatment effect and analysis method. As an approach to provide an interpretable measure of the relative likelihood of having VPI at age 5, following 6-month compared with 12-month surgery, in the form of a relative risk, the dichotomisation of VPC-Sum scores with a prespecified cut-point was not unreasonable. We hope the trial and our subsequent broader analysis serves to encourage debate about the most appropriate way to analyse ordinal data for any given research question, because the use of ordinal scales is common in cleft outcome research.

## The Issue of Estimands

The TOPS statistical analysis strategy referred to the use of estimands, which are more frequently discussed in the context of clinical trials following the recent guidelines published by the International Council for Harmonisation.^24^ The term ‘estimand’ is well established and refers to a clear description of the treatment effect to be quantified in order to answer the research question.^25^ The estimand framework was introduced to improve the handling of post-randomisation events (also known as intercurrent events) that can impact the measurement, and interpretation, of the endpoint in clinical trials.^26^ In the TOPS trial, the post-randomisation event that was acknowledged in the post-hoc analysis was secondary speech surgery, because its indication is the same as the primary outcome (ie, VPI).

The are several possible estimand strategies for dealing with post-randomisation events and two of these were utilised in TOPS. The Treatment Policy Estimand was the primary strategy and corresponds to an Intention to Treat Analysis, by assuming that the post-randomisation event is not relevant to the original question of interest.^26^ This is appealing from a research perspective as it preserves the randomisation and ostensibly addresses the straightforward question of the causal effect of 6-month versus 12-month initial surgery.^27^

A version of the Composite Estimand strategy was used in a post-hoc analysis. This recognises the importance of a post-randomisation intercurrent event and redefines the primary endpoint to incorporate it: in TOPS, the children who had secondary surgery were considered to have reached the same endpoint as those who had VPI at 5 years of age. This is appealing because it recognises secondary speech surgery as an important post-randomisation event but adds further layers of complexity for interpretation. For example, in the context of TOPS, those randomised to 6-month surgery had a greater time period in which to be considered for secondary surgery and there may well also have been additional post-randomisation events that occur before the age of five that could have been considered (such as speech therapy). In effect, the composite estimand strategy represents a refocussing of the trial away from the prespecified primary endpoint at 5 years of age.

The decision to acknowledge the importance of secondary speech surgery within the post-hoc analysis, is in line with an accepted international precedent to consider the occurrence of secondary speech surgery alongside 5-year speech outcomes.^15,21,28–31^ Whether the TOPS analysis should have redefined the endpoint to include secondary surgery as the primary strategy is debateable, but by not mentioning the increased prevalence of secondary surgery in the 6-month group as a caveat in the trial abstract, the interpretation of the trial conclusion is less transparent. Displaying the output from the two estimand strategies, as in Table 2, may have helped the reading audience to see how the two strategies provided different strengths of evidence regarding the primary endpoint.

**Table 2.** The Impact of the Two Estimand Strategies Used in TOPS on the Primary Endpoint, VPC-Sum.

Primary endpoint analysis strategy	VPC-Sum Score	6-month group	12-month group	Risk Ratio	95% CI	P Value
Treatment Policy Estimand (Using the 5-year endpoint with the intention to treat principle)	4–6	21/235 (8.9%)	34/226 (15.0%)	0.59	0.36 to 0.99	0.04
Composite Estimand (Redefining the 5-year endpoint to include occurrence of secondary speech surgery)	4–6	48/235 (17.4%)	50/226 (19.9%)	0.88	0.60 to 1.28	0.50

## Summary and Lessons for Clinical Practice and Future Trials

The TOPS trial was a landmark cleft study and is laudable for its scientific rigour in terms of pre-registration, advance publication of its protocol and standardised data collection. Publication in a high impact factor journal such as the NEJM gives the paper and its headline conclusions gravitas, and this is undoubtedly important for patients and families affected by cleft palate. However, given the statistical uncertainty surrounding the primary outcome and with most of the secondary speech outcomes indicating minimal differences between the two trial arms, we agree with Tse and Jackson in their summary that TOPS did not deliver clear evidence as to whether the benefits of surgery at 6-months outweigh the potential harms.^5^

TOPS provided incredibly rich data, yet any expectations for TOPS to have been able to provide a definitive answer were arguably unfounded, as is the case for many clinical trials in challenging clinical settings. This has been the case in previous surgical trials, which are known to have specific challenges in the way they are conducted and reported.^32,33^ Even in more straightforward settings, the interpretation of trial results should invariably be more nuanced than a simple “up or down” according to a statistical test of a primary null hypothesis. This is not just because of the “fragility” of such tests (to small numbers of cases going in different directions), but also to the under-recognised presence of effect heterogeneity, which implies that there will often be difficult-to-identify subgroups in which treatment effects are stronger or weaker than the headline average effect.

But what then can we learn from TOPS in terms of wider interpretation and application to clinical practice? For the management of infants who fit the inclusion criteria, TOPS provides marginal evidence of reduced VPI at 5 years when surgery is performed at 6 months compared to 12 months. This is useful clinical information because it demonstrates that good speech outcomes are likely to be achieved for most infants at either time point. For countries, such as the United Kingdom, where cleft palate reconstruction is mostly performed at a time that falls between 6 and 12 months, the TOPS data should provide some reassurance.^34,35^ Further data specifically relating to the timing of surgery in more complex patients and in low-and-middle-income settings is required.

It is unclear whether anything like the TOPS trial, in terms of magnitude and effort, will be repeated in future. Randomised controlled trials (RCTs) remain the gold standard for answering research questions regarding treatment effect because of their ability, when carefully conducted, to directly estimate average causal effects by eliminating the threat of confounding bias due to unobserved factors that may differ between comparison groups. International cross-linguistic RCTs should consider pre-specifying adjustments in data analysis for potential imbalances in baseline characteristics such as country of origin and ethnicity, if these are judged to be prognostic for outcome.^36,37^

As an alternative approach to RCTs, large longitudinal observational studies, such as the Cleft Collective,^38^ CORNET Study^39^ and the International Family Study^40^ have shown promising potential in their ability to provide answers for important questions in cleft care. However, valid causal inference from observational data requires strong assumptions, especially regarding the measurement and control of confounding factors. Recent work has highlighted the power of the “target trial” framework for organising such studies.^41^ Under this approach the question of interest is first sharply delineated in terms of a hypothetical “perfect” randomised trial that would answer it, following which one assesses the extent to which emulation of that trial using observational data may be possible, with a range of novel methods available to reduce the threat of biases associated with the observational nature of the data.^42^

We are extremely grateful to the TOPS Trial Team for their openness and support with data sharing and secondary data analysis, and believe this demonstrates a collaborative culture within cleft research that should ensure further advances are made. Perhaps though, one of the lasting legacies of TOPS will be a demonstration that in a complex condition such as orofacial cleft, no one scientific methodology or analysis strategy will be able to provide a definitive answer. Instead, a gradual amalgamation of data from various methodologies with transparent reporting strategies will ultimately lead to an increasingly clearer perspective.^43^

## Supplemental Material

sj-docx-1-cpc-10.1177_10556656241253949 - Supplemental material for Analysis and Reporting of Randomized Trials in Cleft Palate Surgery: Learning from the Timing of Primary Surgery (TOPS) Trial Supplemental material, sj-docx-1-cpc-10.1177_10556656241253949 for Analysis and Reporting of Randomized Trials in Cleft Palate Surgery: Learning from the Timing of Primary Surgery (TOPS) Trial by Matthew Fell, Ginette Phippen, Stephanie van Eeden, David Chong, Marc C. Swan, Simon van Eeden and John B. Carlin in The Cleft Palate Craniofacial Journal

sj-docx-2-cpc-10.1177_10556656241253949 - Supplemental material for Analysis and Reporting of Randomized Trials in Cleft Palate Surgery: Learning from the Timing of Primary Surgery (TOPS) Trial Supplemental material, sj-docx-2-cpc-10.1177_10556656241253949 for Analysis and Reporting of Randomized Trials in Cleft Palate Surgery: Learning from the Timing of Primary Surgery (TOPS) Trial by Matthew Fell, Ginette Phippen, Stephanie van Eeden, David Chong, Marc C. Swan, Simon van Eeden and John B. Carlin in The Cleft Palate Craniofacial Journal
