# Supplementary material for: Analysis and Reporting of Randomized Trials in Cleft Palate Surgery: Learning from the Timing of Primary Surgery (TOPS) Trial
Source: Cleft Palate Craniofac J. 2024 May 9;62(8):1436–42. doi: 10.1177/10556656241253949 (PMC12198463; doi:10.1177/10556656241253949)
Supplement: sj-docx-1-cpc-10.1177_10556656241253949 - Supplemental material for Analysis and Reporting of Randomized Trials in Cleft Palate Surgery: Learning from the Timing of Primary Surgery (TOPS) Trial [file sj-docx-1-cpc-10.1177_10556656241253949.docx]

**Supplementary Data**

**Supplementary Table 1:** VPC Sum ordinal data acquired from the TOPS Trial Group via a data request

| **Trial Arm** | **VPC Sum Score** | **Brazil** | **Denmark** | **Norway** | **Sweden** | **UK** | **Total** |
| --- | --- | --- | --- | --- | --- | --- | --- |
| **6 month** | 0 | 52 | 12 | 5 | 12 | 60 | 141 |
|  | 1 | 10 | 2 | 6 | 7 | 13 | 38 |
|  | 2 | 2 | 3 | 3 | 5 | 8 | 21 |
|  | 3 | 1 | 1 | 3 | 4 | 5 | 14 |
|  | 4 | 1 | 3 | 4 | 0 | 5 | 13 |
|  | 5 | 0 | 0 | 1 | 0 | 2 | 3 |
|  | 6 | 0 | 1 | 0 | 1 | 3 | 5 |
| **12 month** | 0 | 50 | 11 | 6 | 11 | 52 | 130 |
|  | 1 | 4 | 2 | 5 | 8 | 7 | 26 |
|  | 2 | 7 | 2 | 4 | 4 | 7 | 24 |
|  | 3 | 3 | 1 | 2 | 3 | 3 | 12 |
|  | 4 | 5 | 4 | 2 | 3 | 6 | 20 |
|  | 5 | 2 | 0 | 0 | 4 | 3 | 9 |
|  | 6 | 1 | 1 | 2 | 1 | 0 | 5 |
