# Supplementary material for: Analysis and Reporting of Randomized Trials in Cleft Palate Surgery: Learning from the Timing of Primary Surgery (TOPS) Trial
Source: Cleft Palate Craniofac J. 2024 May 9;62(8):1436–42. doi: 10.1177/10556656241253949 (PMC12198463; doi:10.1177/10556656241253949)
Supplement: sj-docx-2-cpc-10.1177_10556656241253949 - Supplemental material for Analysis and Reporting of Randomized Trials in Cleft Palate Surgery: Learning from the Timing of Primary Surgery (TOPS) Trial [file sj-docx-2-cpc-10.1177_10556656241253949.docx]

Stata log for analysis of VPC-Sum Ordinal Data

. use tops, clear

. list, sep(0)

+----------------------+

| trt VPCsum count |

|----------------------|

1. | 1 0 141 |

2. | 1 1 38 |

3. | 1 2 21 |

4. | 1 3 14 |

5. | 1 4 13 |

6. | 1 5 3 |

7. | 1 6 5 |

8. | 0 0 130 |

9. | 0 1 26 |

10. | 0 2 24 |

11. | 0 3 12 |

12. | 0 4 20 |

13. | 0 5 9 |

14. | 0 6 5 |

+----------------------+

.

. tab VPCsum trt [fw=count], col

+-------------------+

| Key |

|-------------------|

| frequency |

| column percentage |

+-------------------+

| trt

VPCsum | 0 1 | Total

-----------+----------------------+----------

0 | 130 141 | 271

| 57.52 60.00 | 58.79

-----------+----------------------+----------

1 | 26 38 | 64

| 11.50 16.17 | 13.88

-----------+----------------------+----------

2 | 24 21 | 45

| 10.62 8.94 | 9.76

-----------+----------------------+----------

3 | 12 14 | 26

| 5.31 5.96 | 5.64

-----------+----------------------+----------

4 | 20 13 | 33

| 8.85 5.53 | 7.16

-----------+----------------------+----------

5 | 9 3 | 12

| 3.98 1.28 | 2.60

-----------+----------------------+----------

6 | 5 5 | 10

| 2.21 2.13 | 2.17

-----------+----------------------+----------

Total | 226 235 | 461

| 100.00 100.00 | 100.00

. * ordinal (proportional odds)

. ologit VPCsum i.trt [fw=count]

Iteration 0: Log likelihood = -618.91398

Iteration 1: Log likelihood = -618.27975

Iteration 2: Log likelihood = -618.27958

Iteration 3: Log likelihood = -618.27958

Ordered logistic regression Number of obs = 461

LR chi2(1) = 1.27

Prob > chi2 = 0.2600

Log likelihood = -618.27958 Pseudo R2 = 0.0010

------------------------------------------------------------------------------

VPCsum | Coefficient Std. err. z P>|z| [95% conf. interval]

-------------+----------------------------------------------------------------

1.trt | -.2043642 .1814693 -1.13 0.260 -.5600374 .151309

-------------+----------------------------------------------------------------

/cut1 | .2487736 .132828 -.0115645 .5091116

/cut2 | .872529 .13928 .5995453 1.145513

/cut3 | 1.442053 .1521448 1.143854 1.740251

/cut4 | 1.896629 .1690825 1.565234 2.228025

/cut5 | 2.893641 .2350385 2.432974 3.354308

/cut6 | 3.709878 .3310485 3.061034 4.358721

------------------------------------------------------------------------------

. ologit, or

Ordered logistic regression Number of obs = 461

LR chi2(1) = 1.27

Prob > chi2 = 0.2600

Log likelihood = -618.27958 Pseudo R2 = 0.0010

------------------------------------------------------------------------------

VPCsum | Odds ratio Std. err. z P>|z| [95% conf. interval]

-------------+----------------------------------------------------------------

1.trt | .8151654 .1479275 -1.13 0.260 .5711877 1.163356

-------------+----------------------------------------------------------------

/cut1 | .2487736 .132828 -.0115645 .5091116

/cut2 | .872529 .13928 .5995453 1.145513

/cut3 | 1.442053 .1521448 1.143854 1.740251

/cut4 | 1.896629 .1690825 1.565234 2.228025

/cut5 | 2.893641 .2350385 2.432974 3.354308

/cut6 | 3.709878 .3310485 3.061034 4.358721

------------------------------------------------------------------------------

Note: Estimates are transformed only in the first equation to odds ratios.

.

. * binary splits

. * 0/1

. csi 94 96 141 130

| Exposed Unexposed | Total

-----------------+------------------------+-----------

Cases | 94 96 | 190

Noncases | 141 130 | 271

-----------------+------------------------+-----------

Total | 235 226 | 461

| |

Risk | .4 .4247788 | .4121475

| |

| Point estimate | [95% conf. interval]

|------------------------+------------------------

Risk difference | -.0247788 | -.1146476 .0650901

Risk ratio | .9416667 | .7571946 1.171081

Prev. frac. ex. | .0583333 | -.1710809 .2428054

Prev. frac. pop | .0297361 |

+-------------------------------------------------

chi2(1) = 0.29 Pr>chi2 = 0.5890

. * 1/2

. csi 56 70 179 156

| Exposed Unexposed | Total

-----------------+------------------------+-----------

Cases | 56 70 | 126

Noncases | 179 156 | 335

-----------------+------------------------+-----------

Total | 235 226 | 461

| |

Risk | .2382979 .3097345 | .2733189

| |

| Point estimate | [95% conf. interval]

|------------------------+------------------------

Risk difference | -.0714366 | -.1526843 .009811

Risk ratio | .7693617 | .5698326 1.038757

Prev. frac. ex. | .2306383 | -.0387567 .4301674

Prev. frac. pop | .1175705 |

+-------------------------------------------------

chi2(1) = 2.96 Pr>chi2 = 0.0853

. * 2/3

. csi 35 46 200 180

| Exposed Unexposed | Total

-----------------+------------------------+-----------

Cases | 35 46 | 81

Noncases | 200 180 | 380

-----------------+------------------------+-----------

Total | 235 226 | 461

| |

Risk | .1489362 .2035398 | .175705

| |

| Point estimate | [95% conf. interval]

|------------------------+------------------------

Risk difference | -.0546037 | -.1240839 .0148766

Risk ratio | .7317299 | .4905412 1.091506

Prev. frac. ex. | .2682701 | -.0915059 .5094588

Prev. frac. pop | .1367537 |

+-------------------------------------------------

chi2(1) = 2.37 Pr>chi2 = 0.1236

. * 3/4

. csi 21 34 214 192

| Exposed Unexposed | Total

-----------------+------------------------+-----------

Cases | 21 34 | 55

Noncases | 214 192 | 406

-----------------+------------------------+-----------

Total | 235 226 | 461

| |

Risk | .0893617 .1504425 | .1193059

| |

| Point estimate | [95% conf. interval]

|------------------------+------------------------

Risk difference | -.0610808 | -.1202642 -.0018973

Risk ratio | .5939925 | .3558304 .9915596

Prev. frac. ex. | .4060075 | .0084404 .6441696

Prev. frac. pop | .206967 |

+-------------------------------------------------

chi2(1) = 4.09 Pr>chi2 = 0.0431

. * 4/5

. csi 8 14 227 212

| Exposed Unexposed | Total

-----------------+------------------------+-----------

Cases | 8 14 | 22

Noncases | 227 212 | 439

-----------------+------------------------+-----------

Total | 235 226 | 461

| |

Risk | .0340426 .0619469 | .0477223

| |

| Point estimate | [95% conf. interval]

|------------------------+------------------------

Risk difference | -.0279043 | -.0669589 .0111502

Risk ratio | .5495441 | .2350592 1.284777

Prev. frac. ex. | .4504559 | -.284777 .7649408

Prev. frac. pop | .229625 |

+-------------------------------------------------

chi2(1) = 1.97 Pr>chi2 = 0.1600

. * 5/6

. csi 5 5 230 221

| Exposed Unexposed | Total

-----------------+------------------------+-----------

Cases | 5 5 | 10

Noncases | 230 221 | 451

-----------------+------------------------+-----------

Total | 235 226 | 461

| |

Risk | .0212766 .0221239 | .021692

| |

| Point estimate | [95% conf. interval]

|------------------------+------------------------

Risk difference | -.0008473 | -.0274581 .0257635

Risk ratio | .9617021 | .2822062 3.277287

Prev. frac. ex. | .0382979 | -2.277287 .7177938

Prev. frac. pop | .0195228 |

+-------------------------------------------------

chi2(1) = 0.00 Pr>chi2 = 0.9502
